# Co-Culture of Hematopoietic Stem/Progenitor Cells with Human Osteblasts Favours Mono/Macrophage Differentiation at the Expense of the Erythroid Lineage

**DOI:** 10.1371/journal.pone.0053496

**Published:** 2013-01-22

**Authors:** Simona Salati, Gina Lisignoli, Cristina Manferdini, Valentina Pennucci, Roberta Zini, Elisa Bianchi, Ruggiero Norfo, Andrea Facchini, Sergio Ferrari, Rossella Manfredini

**Affiliations:** 1 Centre for Regenerative Medicine “Stefano Ferrari”, University of Modena and Reggio Emilia, Modena, Italy; 2 SC Laboratorio di Immunoreumatologia e Rigenerazione Tissutale, Istituto Ortopedico Rizzoli, Bologna, Italy; 3 Laboratorio RAMSES, Istituto Ortopedico Rizzoli, Bologna, Italy; 4 Dipartimento di Scienze Mediche e Chirurgiche, University of Bologna, Bologna, Italy; 5 Life Sciences Department, University of Modena and Reggio Emilia, Modena, Italy; University of Frankfurt - University Hospital Frankfurt, Germany

## Abstract

Hematopoietic stem cells (HSCs) are located in the bone marrow in a specific microenvironment referred as the hematopoietic stem cell niche, where HSCs interact with a variety of stromal cells. Though several components of the stem cell niche have been identified, the regulatory mechanisms through which such components regulate the stem cell fate are still unknown. In order to address this issue, we investigated how osteoblasts (OBs) can affect the molecular and functional phenotype of Hematopoietic Stem/Progenitor Cells (HSPCs) and vice versa. For this purpose, human CD34+ cells were cultured in direct contact with primary human OBs. Our data showed that CD34+ cells cultured with OBs give rise to higher total cell numbers, produce more CFUs and maintain a higher percentage of CD34+CD38- cells compared to control culture. Moreover, clonogenic assay and long-term culture results showed that co-culture with OBs induces a strong increase in mono/macrophage precursors coupled to a decrease in the erythroid ones. Finally, gene expression profiling (GEP) allowed us to study which signalling pathways were activated in the hematopoietic cell fraction and in the stromal cell compartment after coculture. Such analysis allowed us to identify several cytokine-receptor networks, such as WNT pathway, and transcription factors, as TWIST1 and FOXC1, that could be activated by co-culture with OBs and could be responsible for the biological effects reported above. Altogether our results indicate that OBs are able to affect HPSCs on 2 different levels: on one side, they increase the immature progenitor pool *in vitro*, on the other side, they favor the expansion of the mono/macrophage precursors at the expense of the erythroid lineage.

## Introduction

All hematopoietic cells are derived from Hematopoietic Stem Cells (HSCs) which are endowed of two key functions: the self-renewal capacity for the maintenance of a stable HSC pool in the bone marrow (BM) and the differentiation capacity to give rise to mature myeloid and lymphoid cells [1].

HSCs are located in the BM in a specific microenvironment referred as the hematopoietic stem cell niche, which plays a pivotal role in regulating their survival, self-renewal and differentiation. HSCs are retained in a quiescent state in the BM, where they are anchored to specialized niches along the endosteum and in perivascular sites adjacent to the endothelium. In these specific microenvironments, HSCs interact with a variety of stromal cells including fibroblasts, endothelial cells, reticular cells, mesenchymal stem cells, osteoblasts and adipocytes.

Osteoblasts (OBs) are key partecipants providing signals for HSC trafficking, proliferation and survival. In 1994 Taichman RS et al. firstly demonstrated that primary human OBs were able to stimulate the proliferation of primitive CD34+ hematopoietic progenitors *in vitro* [2]. Subsequently, it has been reported that administration of parathyroid hormone (PTH) increases the number of osteoblastic cells in the mouse system, leading to the expansion of HSCs through Notch activation [3].

Communication between HSCs and OBs is essential for HSC self-renewal and proliferation. Human OBs secrete cytokines, such as granulocyte colony-stimulating factor, granulocyte-macrophage colony-stimulating factor, and leukemia inhibitory factor, thus supporting hematopoietic progenitor cell (HPC) function *in vitro* [2], [4], [5].

Furthermore, OBs secrete angiopoietin-1 (Ang-1), thrombopoietin (THPO), and stromal cell-derived factor 1 (SDF-1), which regulate HSC maintenance. In particular, the interaction of Tie2 with its ligand Ang-1 maintains *in vivo* long-term repopulating activity of HSCs, induces HSC adhesion to bone and forces HSCs to become quiescent, suggesting that the Tie2/Ang-1 signaling pathway plays a critical role in the maintenance of mouse HSCs in a quiescent state in the BM [6]. Recently, Yoshihara H. et al. reported that mouse HSCs are in close contact to THPO-producing osteoblastic cells at the endosteal surface in the trabecular bone, suggesting the THPO/MPL pathway as a critical component of the HSC osteoblastic niche [7]. Although several components of the BM niche have been identified, how different cellular elements collaborate to promote HSC self-renewal and to maintain the stem cell pool is still unknown. In addition, the regulatory mechanisms that contribute to the formation of a quiescent or permissive microenvironment for HSC differentiation still need to be identified. More importantly, most of our knowledge on the hematopoietic stem cell niche comes from transgenic mouse models, so it is not clear whether or not these information can be transferred to the human system.

In the present study, we investigated how human primary OBs can affect the molecular and functional phenotype of HSPCs and vice versa in adherent co-culture system. Our data demonstrate that OBs are able to expand the hematopoietic progenitor pool *in vitro* and to induce a strong increase in the clonogenic activity of human HSPCs. Moreover, our results pointed out that OBs are also able to affect the differentiation capacity of CD34+ cells favouring the expansion of the mono/macrophage lineage at the expense of the erythroid and granulocytic ones. Finally, GEP analysis enabled us to identify several cytokine-receptor networks and transcription factors that could be activated by co-culture with OBs and could be responsible for the biological effects observed *in vitro*.

## Methods

### Ethics Statement

Human CD34+ cells were purified upon donor's informed written consent from umbilical Cord Blood (CB) samples, collected after normal deliveries, according to the institutional guidelines for discarded material (Clearance of Ethical Commitee for Human experimentation of Modena: Segretary office Saverio Santachiara, santachiara.saverio@policlinico.mo.it, approval date: 18.01.2005; approval file number # 793/CE).

Human osteoblasts (OBs) were directly isolated from the trabecular bone harvested from the inner portion of the tibia plateau. The study was approved by the local Ethical Committee and informed written consent was obtained from each patient (Ethical Committee of Istituto Ortopedico Rizzoli, Secretary office massimiliano.luppi@ior.it, Approved December 21, 2004, Prot.141/CE/US/ML)

### CD34+ stem/progenitor cell purification

Human CD34+ cells were purified from umbilical cord blood (UCB) samples, as previously described [8]. CD34+ cell purity assessed by flow cytometry was always >95%.

After purification, CD34+ cells were seeded in 24-well plates at 5×10^5^ cells/mL in Iscove's modified Dulbecco's medium (IMDM) (GIBCO, Grand Island, NY, USA) containing 20% Human Serum (Bio-Whittaker, Walkersville, MD, USA), SCF (50 ng/ml), FLT3LG (50 ng/ml), TPO (20 ng/ml), IL-6 (10 ng/ml) and IL-3 (10 ng/ml) (all from R&D Systems, Minneapolis, MN, USA).

### Isolation of human osteoblasts

Human OBs were directly isolated from the trabecular bone harvested from the inner portion of the tibia plateau of 16 patients undergoing total knee replacement for osteoarthritis (mean ± SD age 70±8.2 years) as previously reported [9]. The bone chips were fed with medium twice a week. After 2 weeks, they were removed and the OBs were allowed to grow until confluent and analyzed at the first and second passages.

### Osteoblast proliferation and immunocytochemical analysis

OBs were seeded in 96-well plates (5×10^3^ cell/well) and incubated in 4 different media: CTR: consisting of DMEM/F12 medium supplemented with 20% FBS, COND.1: consisting of a 1∶1 mixture of IMDM and DMEM/F12 supplemented with 20% FBS, SCF 25 ng/ml, FLT3LG 25 ng/ml, TPO 10 ng/ml, IL-6 5 ng/ml and IL-3 5 ng/ml, COND.2: consisting of a 1∶1 mixture of RPMI and DMEM/F12 supplemented with 20% FBS, SCF 25 ng/ml, FLT3LG 25 ng/ml, TPO 10 ng/ml, IL-6 5 ng/ml and IL-3 5 ng/ml, COND.3: consisting of a 1∶1 mixture of IMDM and DMEM/F12 supplemented with 20% FBS, SCF 5 ng/ml, FLT3LG 5 ng/ml, TPO 2 ng/ml, IL-6 1 ng/ml and IL-3 1 ng/ml. After 72 h, ^3^H-Thymidine (Amersham Pharmacia Biotech Italia, Milan, Italy) was added at a final concentration of 2 µCi/ml for 18 h at 37°C. At the end of the incubation time, the 96-well plates were aspirated onto fiberglass filters using a Filtermate 196 Harvester (Packard Instrument Company, Meriden, CT, USA). The filters were then dried and bound radioactivity was assayed by liquid scintillation counting using a Top Count Microplate Scintillation Counter (Packard Instrument Company). Data were expressed as counts per minute± S.D.

For immunocytochemical analysis OBs (1×10^4^/well) were seeded in 8-well chamber slides and after 72 h fixed in 4% PFA for 20 minutes at room temperature (RT) and then hydrated with TBS 1% BSA for 5 minutes at RT. Cells were then incubated with the following anti-human monoclonal antibodies: anti-RUNX-2, anti-osteocalcin (R&D system, Minneapolis, MN, USA), and anti-alkaline phosphatase (AP) (DSHB Department of Biological Sciences, iowa City, IA, USA), for 1 hour at RT. The slides were washed three times with TBS 1% BSA and then sequentially incubated with multilinker biotinylated secondary antibody and alkaline phosphatase-conjugated streptavidin (Biocaremedical, Concord, CA, USA) at RT for 20 minutes. The slides were developed using fast red as a substrate, counterstained with haematoxylin, mounted with glycerol jelly and evaluated in a brightfield microscope. Negative and isotype matched controls were performed.

### Co-culture condition

24 h after isolation, CD34+ cells were seeded directly onto semiconfluent OB monolayers at a final density of 7×10^3^ cell/cm^2^ in 6-well plates. Cultures were maintained for 2 weeks in medium consisting of a 1∶1 mix of IMDM and DMEM/F12 (Dulbecco's Modified Eagle Medium: Nutrient Mixture F-12, both Celbio, Italy) supplemented with 20% FBS (Lonza, Italy), SCF 5 ng/mL, FLT3LG 5 ng/mL, TPO 2 ng/mL, IL-3 1 ng/mL and IL-6 1 ng/mL (all R&D Systems, Minneapolis, MN, USA), streptomicin 100 µg/mL, penicillin 100 µg/mL and L-glutamine 2 mM (all purchased from Celbio, Italy). In order to better assess erythroid and megakaryocytic differentiation, cells were cultured in serum free medium consisting of a 1∶1 mix of of IMDM and DMEM/F12 supplemented with 20% BIT (StemCell Technologies, Vancouver, Canada), SCF 5 ng/mL, FLT3LG 5 ng/mL, TPO 1 ng/mL, IL-6 1 ng/mL (all R&D Systems, Minneapolis, MN, USA), streptomicin 100 µg/mL, penicillin 100 µg/mL and L-glutamine 2 mM (all Celbio, Italy). Cells were harvested by trypsinization and vigorous pipetting on days 3, 7, 10 and 14 of co-culture.

### Isolation of CD34+ cells and osteoblasts from co-cultured samples

The separation of CD34+ cells from OBs was performed using a magnetic cell sorting procedure (EasySep Human CD34+ positive selection kit, StemCell Techonologies Inc.; Vancouver, Canada). The fraction of CD34+ cells that was not cultivated on OB layers (CD34+ CTR) was sorted using the same parameters to exclude differences in clonogenic activity and gene expression due to a different treatment. CD34+ cell purity assessed by flow cytometry was >95% for both co-cultured and control samples. On the other hand, OBs were purified from the hematopoietic cells based on the expression of the pan-leukocyte marker CD45. CD45-negative cells were isolated by means of negative-selection magnetic beads sorting from the cells collected after co-culture. Purity of OBs preparations was assessed firstly by flow cytometric analysis of CD45 expression and subsequently by immunocytochemical staining of OB-specific markers, such as RUNX-2, Osteocalcin, Alkaline Phosphatase and Collagen type I as described above. The percentage of positive cells was measured on 10 RGD images acquired for each marker by image analysis with Software NIS Element (Eclipse 90 I, Nikon) using an objective at 10× magnification.

### Immunophenotypic analysis

Differentiation of CD34+ cells was monitored by flow cytometric analysis (BD FACSCanto II, Becton Dickinson, USA) of CD14, CD34, CD38, CD41, CD66b, CD163, MPO, and GPA expression, performed at days 3, 7,10 and 14 of culture, as previously described [10]. The values relative to immunophenotypic analysis are reported as ± 2S.E.M from ten independent experiments.

### Immunofluorescent staining

Cytospins of CD34+ cells were fixed with 4% paraformaldehyde (PFA) and permeabilized using 0.3% Triton X-100 in PBS for 10 minutes. After blocking with 3% BSA in PBS, slides were incubated with rabbit monoclonal anti-human collagen type I (COL1A1) antibody (Invitrogen, Carlsbad, CA) at 1∶500 dilution and with mouse monoclonal anti-human CD34 antibody (Becton Dickinson, BD) at 1∶50 dilution for 1 h at 37°C. This was followed by incubation with goat anti-rabbit Alexa Fluor 568-conjugated and goat anti-mouse Alexa Fluor 488-conjugated secondary antibodies (Invitrogen, Carlsbad, CA) at 1∶2000 dilution for 1 h at 37°C. All incubations were followed by 3 washes with PBS solution. Nuclear counterstaining was performed with 4′,6-diamino-2-phenylindole (DAPI). The slides were mounted with Vectashield Mounting Medium (Vector Laboratory Inc). Finally, fluorescence imaging was performed using the Zeiss LSM 510 Meta Confocal Microscope (Zeiss, Germany) and digital images of representative areas were taken. To ensure random sampling, 50 images/slide were captured and cells positive for Col1a1 were scored.

### Methylcellulose clonogenic assay

Human Colony Forming Cells (CFUs) were cultured in methylcellulose as previously described [11]. After 14 days of culture at 37°C in a humidified atmosphere with 5% CO_2_, erythroid burst-forming units (BFU-E), erythroid colony-forming units (CFU-E), colony-forming units granulocyte (CFU-G), macrophage (CFU-M), granulocyte-macrophage (CFU-GM), and colony-forming units granulocyte/erythrocyte/macrophage/megakaryocyte (CFU-GEMM) were scored. The values are reported as ± 2S.E.M from five independent experiments.

### RNA extraction and microarray analysis

Total cellular RNA was extracted after 3 days of treatment from 0.3×10^6^ cells of each sample using RNeasy Micro kit (Qiagen, Valencia, CA) following the protocol supplied by the manufacturer. For both CD34+ cells and OBs, total RNA pools (100 ng) of Control (CTR) and co-culture (COCULT) cells, obtained from 5 independent experiments, were converted in biotinilated aRNA according to the GeneChip 3′ IVT Express Kit protocol advised by Affymetrix. Similarly, the Affymetrix Human HG-U133plus2 GeneChip arrays hybridization, staining, and scanning, were performed using Affymetrix standard protocols (Affymetrix, Santa Clara,CA) as previously described [10].

The GeneChip Operating Software (GCOS) absolute analysis algorithm was used to determine the amount of a transcript mRNA (Signal), while the GCOS comparison analysis algorithm was used in order to compare gene expression levels between two samples.

Differentially expressed genes were selected as the sequences showing a Change call “I” or “D” and Signal Log Ratio ≥1 or ≤−1 in the pair-wise comparisons between COCULT and CTR cells.

All the data have been deposited in the Gene Expression Omnibus MIAME compliant public database, at http://www.ncbi.nlm.nih.gov/geo/query/acc.cgi?acc=GSE38091.

DAVID 6.7 (National Institute of Allergy and Infectious Diseases, http://david.abcc.ncifcrf.gov/) was used to examine selected lists of genes in order to identify over-representation of functional categories according with gene-ontology classification. To validate microarray data, a set of modulated genes was monitored by quantitative real-time (QRT) PCR as previously described [8], for assay IDs see **Table 1**.

**Table 1:** Assay IDs for quantitative real-time (QRT) PCR.

Assay ID	Context sequence	Gene ID
Hs00174092_m1	TGCTGCTGAAGGAGATGCCTGAGAT	IL1A
Hs01555410_m1	GATGAAGTGCTCCTTCCAGGACCTG	IL1B
Hs99999148_m1	TCCAGCGCTCTCAGCACCAATGGGC	CCL4
Hs01011368_m1	TATTGTGCGTCTCCTCAGTAAAAAA	CCL20
Hs00171022_m1	CCTTCAGATTGTAGCCCGGCTGAAG	CXCL12
Hs01090305_m1	CGGCCTGCCAGATGTGCCGGTGACT	GAS6
Hs00559473_s1	CCTTCCCTTCCAGCCAGTCTCTGTA	FOXC1
Hs00361186_m1	GCCGGAGACCTAGATGTCATTGTTT	TWIST1
Hs00234140_m1	CTCGCTCAGCCAGATGCAATCAATG	CCL2
Hs00236937_m1	CTGAACAGTGACAAATCCAACTGAC	CXCL1
Hs00171455_m1	GTCTTGGCGGCAGGAGTTGTGCCCC	LIF
Hs00601975_m1	GCTGAAAAATGGCAAATCCAACTGA	CXCL2
Hs00164004_m1	CCTCGACTTGGCCTTCCTCTTGG	COL1A1

### Statistical analysis

The statistics used for data analysis (CD34+ COCULT vs CD34+ CTR) was based on the two-tail t-Student test for averages comparison in paired samples. Data were analyzed by Microsoft Excel Software (version 2007) and *p*<0.05 was considered significant.

## Results

### OBs support Hematopoietic Stem Progenitor Cell (HPSC) expansion and function

CD34+ cells from UCB were cultured for 14 days in either liquid culture or over a monolayer of human primary OBs isolated from the trabecular bone. Co-cultures of OBs and HPCs were initiated (day 0) 24 hours after CD34+ cells isolation from UCB by seeding 7×10^3^ CD34+ cells/cm^2^ on a subconfluent monolayer of OBs. Cultures were supplemented with exogenous cytokines as detailed in Material and Methods.

In preliminary experiments, multiple mixes of media and decreasing doses of hematopoietic cytokines were tested in order to determine the mix of media capable of supporting both osteoblasts and CD34+ cells without modifying cell growth and differentiation properties (**Figure S1, S2 and S3**).

**Figure 1:**
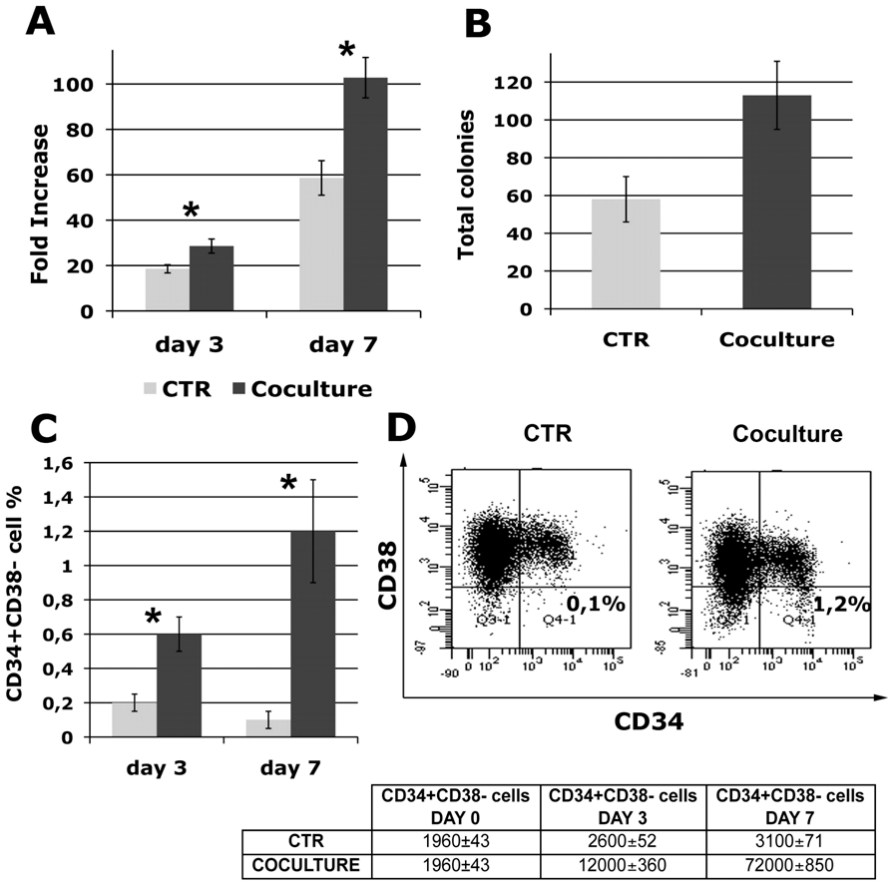
Co-culture with OBs supports hematopoietic stem and progenitor cell function. 7×10^3^ CD34+ cells were cultured alone or in presence of freshly prepared OBs for 3 and 7 days and the following parameters were measured: (A) Fold increase in total cell number calculated from the original CD34+ cells seeded at day 0. (B) Total CFU output for Control and Co-culture CD34+ cells, cells were plated at day 3 of culture and colonies were scored 14 days after plating; (C) percentage of CD34+CD38- cells; and (D) percentage of CD34+CD38- cell population present in Control and Co-cultured samples at day 7, representative flow cytometry is shown. Absolute numbers of CD34+CD38- cells at day 3 and day 7 of co-culture are shown in table. Values are reported as mean ± 2SEM., * = *p*<0.05 versus Control. Abbreviation: CTR, control.

First of all, we investigated the ability of human primary OBs to support hematopoietic stem/progenitor cells in culture. After 3 and 7 days, fold increase in total cell number generated from CD34+ cells, isolated as described in Material and Methods from co-culture and Control samples, was significantly (p≤0.05) higher in co-culture compared with control liquid culture (28,6±3,1 vs 18,57±1,8-fold at day 3, 102,8±8,9 vs 58,61±7,6-fold at day 7) (**Figure 1A**). CD34+ cells were also assessed for their colony-forming ability in methylcellulose-based assay. The number of CFUs generated by CD34+ cells increased by 2±0,4 fold when CD34+ cells were cultured with OBs as compared to being cultured alone (**Figure 1B**). The progeny of CD34+ cells was also assessed for CD34 and CD38 antigen expression at day 3 and 7 of culture. The percentage of CD34+CD38- cells was significantly (p≤0.05) higher in the co-culture compared to the control sample (0,6±0,1% vs 0,2±0,05% at day 3, 1,2±0,3% vs 0,1±0,05% at day 7) (**Figure 1C and 1D**), suggesting that the maintenance of CD34+CD38- cells may be responsible for the increase in CFU output of co-cultured HPCs. This increase in the percentage of CD34+CD38- cells translates into a 5-fold increase and a 23-fold increase at day 3 and 7 respectively, if we take into account the raise in total cell number generated by CD34+ cells co-cultured with OBs (absolute numbers of CD34+CD38- cells at day 3 and day 7 of co-culture are shown in table in **Figure 1**).

Taken together, the increase in total cell number, the higher number of clonogenic cells and the increased percentage of CD34+CD38- cells suggest that OBs are able to enhance HSPC properties, by promoting their expansion while maintaining a primitive phenotype.

### Impact of OBs on HPSC differentiation capacity

**Figure 2:**
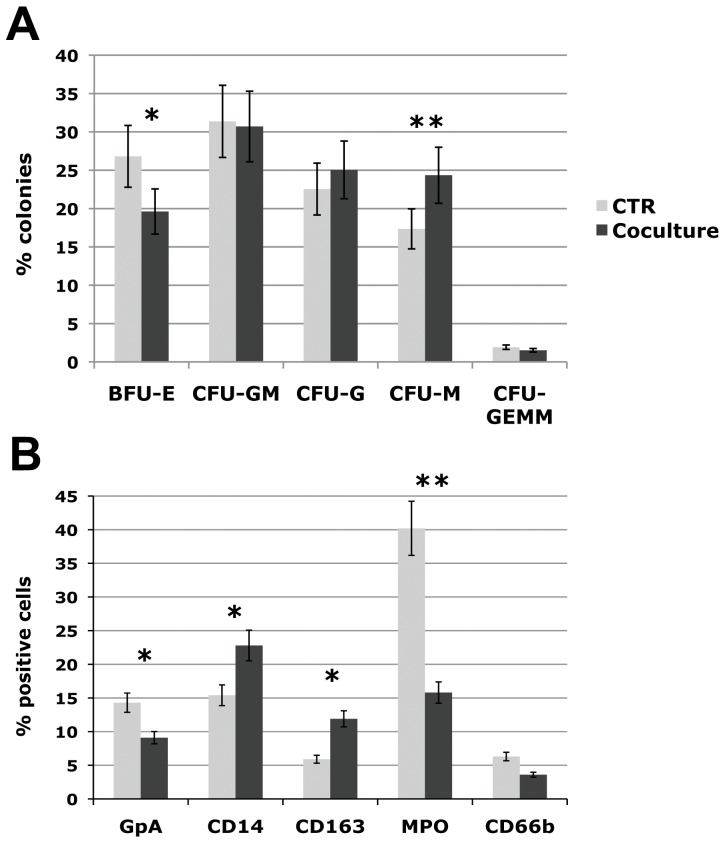
Differentiation capacity of CTR and co-cultured CD34+ cells. (A) Results of statistical analysis of methylcellulose-based clonogenic assay performed on CD34+ cells plated after 3 days of co-culture with human OBs and scored after 14 days. (A) Count of different colony types; (B) Results of statistical analysis on the percentage of cells positive for hematopoietic lineage differentiation markers (GPA, CD66b, MPO, CD14, and CD163) performed by flow cytometry at day 10 and 14 of co-culture. Values are reported as mean ± 2S.E.M., * = *p*≤0.05 vs CTR, ** = *p*≤0.001 vs CTR. The results come from five independent experiments. Abbreviations: BFU-E, Burst forming unit-erythroid; CFU, Colony forming unit; E, erythrocyte; G, granulocyte; M, monocyte; GM, granulocyte-monocyte; GEMM, granulocyte-erythrocyte-monocyte-megakaryocyte.

To better characterize the effects of OBs on stem/progenitor cell differentiation, CD34+ cells purified from control and co-culture samples at day 3 were plated in methylcellulose-based medium in a set of five independent experiments. As described above, clonogenic assay results demonstrated that the clonogenic capacity of CD34+ cells co-cultured with OBs was increased by 2 fold (**Figure 1B–C**). Interestingly, the methylcellulose-based clonogenic assay showed a significant increase (p≤0.001) of the percentage of monocyte (CFU-M) colonies in OB-co-cultured CD34+ cells coupled to a decrease of the erythroid ones (BFU-E), while granulocytic progenitors (CFU-G) were not significantly affected (**Figure 2A**).

The effects of OBs on CD34+ cells were also evaluated in long-term co-culture. CD34+ cells were seeded at 7×10^3^ cell/cm^2^ on a monolayer of primary human OBs. At day 10 and 14 of co-culture, the cells were harvested by trypsinization and vigorous pipetting in order to dissociate hematopoietic cells strictly adherent to OBs and analyzed for the expression of lineage differentiation markers. Flow cytometric analysis was performed at day 10 on GPA and CD41, respectively erythroid and megakaryocytic markers, and at day 14 on the mono/macrophage markers CD14 and CD163, and on the granulocytic markers MPO and CD66b (**Figure 2B**). Our data showed an increase in the percentage of cells positive for the mono/macrophage markers coupled to a decrease in the percentage of cells positive for the granulocytic and erythroid markers, whereas the megakaryocytic marker CD41 was not modulated (data not shown). Overall, these results indicate that co-culture with OBs induces a strong expansion of the mono/macrophage lineage coupled to a decrease in the percentage of erythroid-committed cells.

### Gene Expression Profile (GEP) of CD34+ cells co-cultured with OBs

Next, we investigated the changes in gene expression induced in HPCs by co-culture with primary human OBs, using Affymetrix HGU133Plus2 GeneChip array. Microarray analysis was performed on CD34+ cells isolated at day 3 of co-culture based on the observation that such timing was sufficient to exert a significant modulation of HSPC clonogenic and differentiation capacity in methylcellulose-based assay.

Using the filtering procedure described in Material and Methods, we identified a list of genes significantly modulated in CD34+ cells by OB coculture (486 probesets increased, 110 probesets decreased in CD34+COCULT versus CD34+Control). DAVID 6.7 analysis showed that the “Positive regulation of cell proliferation”, “Cell differentiation”, “Regulation of Wnt receptor signaling pathway” GO categories are mainly represented in the gene list of probesets increased in CD34+COCULT vs CD34+Control cells (**Table S1A**). Consistently with the adhesion of the HSPCs to the osteoblastic layer observed in co-culture, the GO categories “Cell Adhesion” and “Extracellular matrix organization” are significantly represented in the list of increased genes. Conversely, GO categories down-regulated by coculture with OBs include “Oxygen transport” and “Erythrocyte differentiation” (**Table S1B**), in agreement with the down-regulation of the erythroid development reported above.

**Figure 3:**
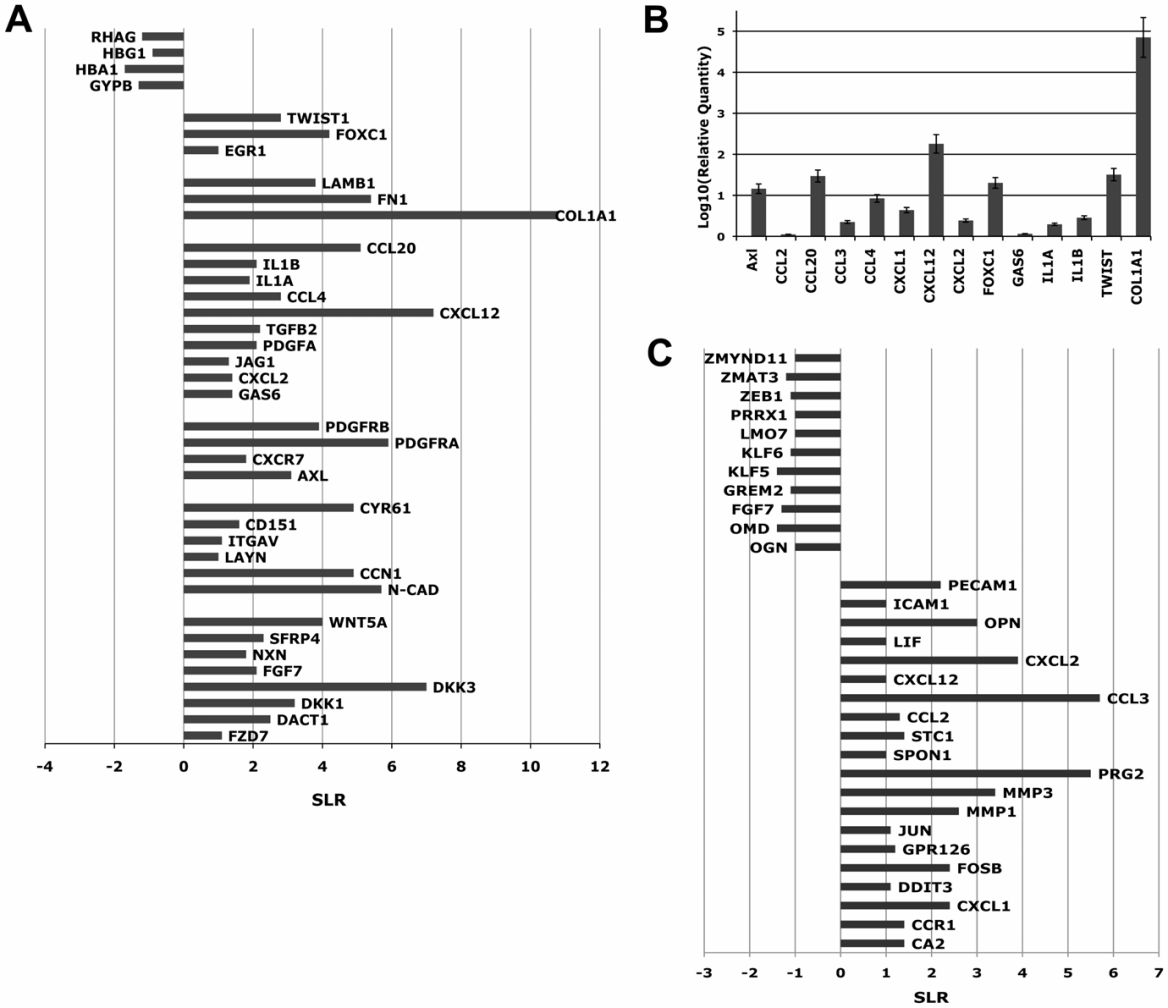
Changes in gene expression profile induced by co-culture in OBs and CD34+ cells. (A) Microarrays analysis was performed at day 3 of co-culture on pooled RNAs from Control and co-cultured CD34+ cells from five independent experiments. Changes in gene expression are reported on the x-axis as signal log ratio (SLR). (B) Expression levels of 13 genes selected as differentially expressed from microarrays data. Gene expression levels were measured by Real-Time Quantitative Polymerase Chain Reaction PCR (RTQPCR) starting from total RNA and were expressed as log10 of Relative Quantity. Results come from five independent experiments. (C) Results of microarrays analysis performed at day 3 of co-culture on pooled RNAs from Control and co-cultured OBs from five independent experiments. Changes in gene expression are reported on the x-axis as signal log ratio (SLR).

As shown in **Figure 3A**, detailed analysis of microarray data showed the up-regulation of several genes involved in the OB-mediated maintenance of hematopoietic stem/progenitor cells such as components of the WNT pathway [12] (WNT5a, FZD7, DACT1, DKK1, DKK3, and SFRP4) [13]. GEP analysis revealed also the up-regulation of genes taking part in HSC adhesion to the BM niche, such as N-cadherin (N-CAD) [14], [15] and LAYN, a newly identified hyaluronan receptor [16]. Moreover, co-culture with OBs induces the up-regulation of Jagged1 (JAG1) [17], GAS6 and its receptor Axl [18], which are involved in the maintenance of HSCs in a quiescent state. Among up-regulated genes were also found several chemokines and pro-inflammatory cytokines, such as CCL4, IL1A, IL1B and CCL20, that, as already reported by Majka M. et al. [19], can be secreted by hematopoietic precursors and regulate with an autocrine and/or paracrin mechanism the various stages of hematopoiesis. Moreover, GEP analysis showed a strong increase in the expression level of several extracellular matrix components such as, collagen type I (COL1A1), fibronectin 1 (FN1) and laminin-β1 (LAMB1) in CD34+ co-cultured cells.

Finally, some transcription factors that could play a role in the regulation of HSCs survival and maintenance, such as Egr1 [20], Foxc1 [21] and Twist1 [22], were similarly increased in CD34+ co-cultured cells vs control.

On the other hand, among down-regulated genes, we found several genes coding for proteins involved in erythroid differentiation, such as Glycophorin B (GYPB) [23], globin genes (HBA1) and Rh-associated glycoprotein (RHAG) [24] (**Figure 3A**).

In order to confirm microarray data, we carried out a TaqMan QRTPCR analysis on a validation set selected among the differentially expressed genes between CD34+COCULT versus CTR sample. All the assessed genes showed in QRT-PCR the same expression pattern obtained by microarray analysis (**Figure 3B**).

**Figure 4:**
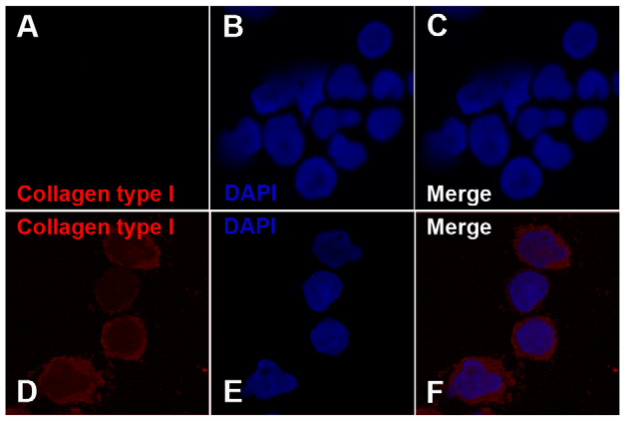
Up-regulation of collagen type I in CD34+ co-cultured cells. Immunofluorescence analysis of collagen type I on human CD34+ cells grown without (Control) or with human primary osteoblasts. Cells were labelled with anti-collagen type I antibody (red fluorescence) and nuclear counterstaining was performed with DAPI (blue fluorescence). In the upper panels, absence of collagen type I expression in CD34+Control cells is shown (A and C). In the lower panels, expression of collagen type I in CD34+ Co-culture (D and F) is shown. Col1A1 is absent on Control CD34+ cells, whereas it is present on the 92±1,3% of Co-culture CD34+ cells.

**Figure 5:**
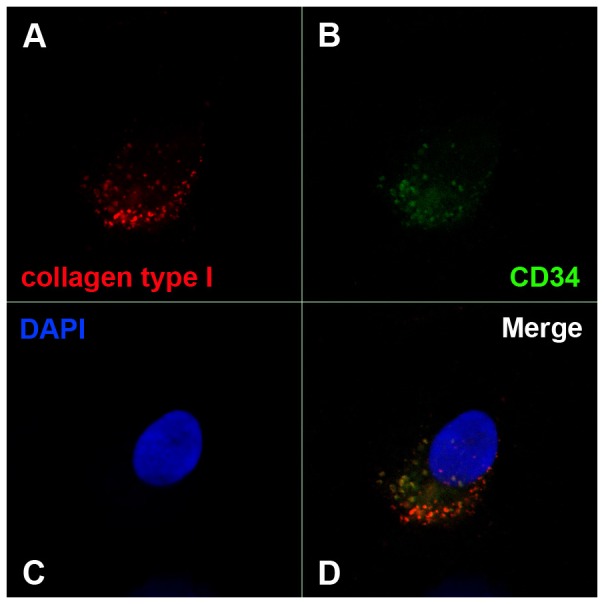
Co-expression of collagen type I and CD34 antigen on CD34+ co-cultured cells. Immunofluorescence analysis of collagen type I on human CD34+ cells grown in co-culture with human primary osteoblasts. Cells were labelled with anti-collagen type I antibody (red fluorescence) and with anti-human CD34 antibody (green fluorescence); nuclear counterstaining was performed with DAPI (blue fluorescence). Co-expression of collagen type I and CD34 antigen was assessed, evaluating the presence of green and red fluorescence in the same cell (D).

In order to rule out the possibility of OBs contamination in the CD34+ cell fraction that could affect GEP results, immunofluorescence (IF) analysis for collagen type I was performed on CD34+ cells purified after co-culture (**Figure S4**). Collagen type I was chosen as it represents a specific marker for OBs [25] and, at the same time, it's strongly up-regulated in CD34+ cells after co-culture. IF confirmed the up-regulation of collagen type I in CD34+ co-cultured cells compared to control (**Figure 4**) strongly suggesting that collagen type I and CD34 antigen are expressed on the same cell (**Figure 5**) and excluding the possibility that the up-regulation observed by GEP analysis was due to OBs contamination of the CD34+ cell preparation.

### Gene Expression Profile (GEP) of OBs co-cultured with CD34+ cells

In order to investigate the changes induced in OBs by co-culture with human HSPCs, we performed GEP analysis on control and co-cultured OBs by means of Affymetrix HGU133Plus2 GeneChip array. OBs were isolated at day 3 of co-culture as described in Material and Methods. Purity of OBs cell preparation was examined both by flow cytometric analysis and immunocytochemical staining for OBs-specific markers. Results, shown in **Figure S5 A–D**, demonstrate that OBs, purified by magnetic bead sorting based on the lack of expression of the CD45 antigen, are in fact 92% negative for CD45 while being positive for several OB-specific markers, such as RUNX-2, Osteocalcin, Alkaline Phosphatase (AP) and Collagen type I (COL1A1) (**Figure S5E**).

Using the filtering procedure described in Material and Methods, we identified a list of genes significantly modulated in OBs after co-culture with CD34+ cells (198 probesets increased, 125 probesets decreased in OB COCULT versus OB Control).

Detailed analysis of differentially expressed genes pointed out the increased expression in OBs co-cultured cells of transcripts coding for cytokines and chemokines involved in the maintenance of HSCs in a quiescent state, such as CCL2 [26], CCL3 [27] and CXCL2 [28] (**Figure 3C**). Interestingly, co-cultured OBs also showed the up-regulation of CXCL12, which is responsible for HSC migration and retention in the BM niche [29] and of Osteopontin (OPN), which is a hematopoietic stem cell niche component that negatively regulates stem cell pool size [30]. Finally, worth of notice is the up-regulation of several adhesion molecules, such as ICAM1 [31] and PECAM1 [32], that could mediate HSPC adhesion to osteoblast cells.

## Discussion

Self-renewal and differentiation divisions of HSCs are tightly regulated by the stem cell niche in the bone marrow. The balance between differentiation and maintenance of the stem cell pool is regulated by complex molecular mechanisms involving a large number of soluble factors and cell-cell interactions. BM stromal cells are key participants of the regulatory machinery governing hematopoietic development. HSCs and osteoblasts are both located near the endosteal surface suggesting a functional relationship between these two cellular populations. Several authors reported that depletion of OBs causes mobilization of hematopoietic stem and progenitor cells to the spleen [33] and, on the other hand, an increase in OB numbers induces a simultaneous increase in HSCs in the BM [34]. In this paper, we sought to examine which regulatory mechanisms are able to regulate the balance between self-renewal and differentiation in the endosteal niche. In order to address this issue we set up a co-culture system composed of human CD34+ cells purified from the CB and human trabecular OBs. Our results demonstrate that co-culture with OBs induces a 23-fold expansion of the hematopoietic progenitor pool and a strong increase in the clonogenic capacity of CB CD34+ cells. Moreover, our data pointed out for the first time the ability of osteoblast cells to affect HSPC differentiation by favoring the expansion of the mono/macrophage lineage at the expense of the erythroid one.

In the presence of OBs, CD34+ cells gave rise to higher total cell numbers, produced more CFUs and maintain a higher percentage of CD34+CD38- cells compared to control culture.

The percentage of CD34+CD38- cells was significantly (p≤0.05) higher in the co-culture compared to the control sample (**Figure 1C**), translating into a 5-fold increase and a 23-fold increase, at day 3 and 7 respectively, if we take into account the raise in total cell number generated by CD34+ cells co-cultured with OBs. At the same time, co-culture with OBs induces a strong increase in the clonogenic capacity of CD34+ cells (**Figure 1B**), suggesting that the maintenance of CD34+CD38- cells may be responsible for the increase in CFU output of co-cultured HPSCs.

Taken together, our results suggest that OBs are able to enhance HPC properties, by promoting their expansion while maintaining a primitive phenotype.

Several authors already reported the ability of OBs to support hematopoietic stem/progenitor cell functions, such as self-renewal and long-term repopulating capacity [35], [36], but to our knowledge, no data is available regarding the capacity of OBs to affect HPC differentiation capacity.

In order to dissect this issue, CD34+ cells purified after 3 days of co-culture with OBs were plated in methylcellulose-based medium and colony numbers and types were scored at day 14. Clonogenic assay results showed a significant increase (p≤0.001) of the percentage of macrophage (CFU-M) colonies in OB-co-cultured CD34+ cells coupled to a decrease of the erythroid ones (BFU-E) (**Figure 2A**). The effects of OBs on HPSCs were also evaluated in long-term co-culture. Flow cytometric analysis of lineage differentiation markers showed an increase in the percentage of cells positive for the mono/macrophage markers CD14 and CD163 coupled to a decrease in the percentage of cells positive for the granulocytic (MPO, CD66b) and erythroid (GPA) markers (**Figure 2B**). Overall, these results indicate that OBs support the expansion of mono/macrophage committed cells while inhibiting the erythroid precursors.

At the aim of characterizing the molecular mechanisms responsible for the biological effects reported above, Gene Expression Profiling (GEP) of CD34+ CTR and CD34+ co-cultured cells was performed. It is important to notice that GEP analysis was performed on highly purified cell populations, as shown in **Figures S4 and S5**. Previous reports attempting to study gene expression profile of hematopoietic cells after culture on stromal layers were unable to separate the hematopoietic cell fraction from the adherent osteoblast cells, consequently the results obtained were strongly compromised by the contamination of the stromal component in the total RNA preparation [36]. In this paper, we were able to separate the CD34+ cells from the remaining osteoblasts and hematopoietic cells, in order to study the changes in gene expression profile induced in HPSCs by the co-culture with human OBs. Our microarray data showed the up-regulation in CD34+ co-cultured cells of several genes already reported as key players in the maintenance of hematopoietic stem/progenitor cell in the BM (**Figure 3A**), such as components of the WNT pathway [12] (WNT5a, FZD7, DACT1, DKK1, DKK3, and SFRP4) and the adhesion molecule N-Cad, that has been shown to regulate HSCs adhesion to the bone lining osteoblasts [37]. Microarray data highlighted not only previously reported components of the osteoblastic stem cell niche, but also showed the up-regulation of a number of genes, not previously described in the context of the BM niche, that could be involved in the signaling network between osteoblasts and HPSCs. For example, the adhesion molecule LAYN, a newly identified hyaluronan receptor [16], could mediate HSPC adhesion to the BM niche, or the signaling network GAS6-Axl, up-regulated after co-culture, could support hematopoietic activity by maintaining HSPCs in a quiescent state [18]. Moreover, GEP analysis showed a strong increase in the expression level of transcription factors that could play a role in the regulation of HSPC survival and maintenance, such as Egr1, Foxc1 and Twist1. Egr1 has been recently described as transcriptional regulator that normally functions to promote HSC quiescence and retention in the BM niche [20]. Foxc1 is a member of the Forkhead/Fox transcription factor family, mainly expressed in mesodermal and neural crest derivatives during development [38], whose expression and function in the hematopoietic system has never been described. Recently, Bloushtain-Qimron N. et al. reported that constitutive expression of Foxc1 in differentiated mammary epithelial cells results in the conversion of the differentiated epithelial phenotype into a progenitor-like phenotype [21]. Thus, the up-regulation of Foxc1 in HSPCs after co-culture with human OBs could be involved in the maintenance of the primitive progenitor phenotype observed *in vitro*.

Twist1 is a highly conserved basic helix-loop-helix transcription factor involved in several pathways that control tumor growth, apoptosis, differentiation, and epithelial–mesenchymal transition [39], [40]. Twist1 directly interacts with and opposes p53 function [41], up-regulation of Twist1 has been reported in several human cancers [42], [43], [44] suggesting that Twist1 functions as an oncogene. In our culture system, Twist1 up-regulation in CD34+ co-cultured cells could favour HSPC survival and this could account, at least in part, for the increase in total cell numbers and CFU output described above.

Conversely, GEP analysis revealed the down-regulation of several genes coding for protein involved in erythroid differentiation, such as Glycophorin B (GYPB) [23], globin genes (HBA1) and Rh-associated glycoprotein (RHAG) [24] (**Figure 3A**). This down-modulation is in agreement with the reduction in erythroid precursors observed both in liquid and semisolid culture.

On the other hand, GEP analysis performed on OBs purified after co-culture revealed the up-regulation of several genes involved in the regulation of the BM stem cell pool size, such as CCL2 (26), CCL3 [27], CXCL2 [28] and OPN [30], [45], and of genes mediating HSC adhesion and retention in the BM stem niche, such as CXCL12 [29], ICAM1 [31] and PECAM1 [32].

Altogether our results indicate that osteoblasts support and influence both maintenance and later differentiation of HSPCs. In the short-term culture, our data demonstrate that OBs are able to enhance HSPC clonogenic capacity and to expand hematopoietic progenitors pool. On the other hand, in the long-term, osteoblasts are able to affect HSPC differentiation, by favoring the mono/macrophage lineage and inhibiting the erythroid one. Moreover, our microarray data allowed us to identify specific signaling pathways and regulatory networks involved in HSPC-osteoblast interaction that could account for the biological effects exerted by OBs on CD34+ cells. In particular, GEP analysis pointed out the up-regulation of several transcription factors, such as TWIST1 and FOXC1, that could play a critical role in HSPC self-renewal and maintenance. Taken together, our data allows us to get new insights on the molecular mechanisms underlying HSPC maintenance and differentiation in the osteoblastic stem cell niche.

## Supporting Information

Figure S1 **Hematopoietic cell culture conditions testing.** Multiple mixes of media and decreasing doses of hematopoietic cytokines were tested in order to determine the mix of media capable of supporting CD34+ cells without modifying cell growth and differentiation properties. 24 h after isolation, CD34+ cells were seeded at a final density of 7×10^3^ cell/cm^2^ in 6-well plates. Cultures were maintained for 2 weeks in 4 different culture conditions: CTR: consisting of Iscove's modified Dulbecco's medium (IMDM) supplemented with 20% FBS, SCF (50 ng/ml), FLT3LG (50 ng/ml), TPO (20 ng/ml), IL-6 (10 ng/ml) and IL-3 (10 ng/ml); COND.1: consisting of a 1∶1 mixture of IMDM and DMEM/F12 supplemented with 20% FBS, SCF (25 ng/ml), FLT3LG (25 ng/ml), TPO (10 ng/ml), IL-6 (5 ng/ml) and IL-3 (5 ng/ml), COND.2: consisting of a 1∶1 mixture of RPMI and DMEM/F12 supplemented with 20% FBS, SCF (25 ng/ml), FLT3LG (25 ng/ml), TPO (10 ng/ml), IL-6 (5 ng/ml) and IL-3 (5 ng/ml), COND.3: consisting of a 1∶1 mixture of IMDM and DMEM/F12 supplemented with 20% FBS, SCF (5 ng/ml), FLT3LG (5 ng/ml), TPO (2 ng/ml), IL-6 (1 ng/ml) and IL-3 (1 ng/ml). Cell cycle analysis, performed at day 3 (A) and 7 (B) of culture by propidium iodide staining, showed no difference between the 3 culture conditions tested and the CTR sample. Flow cytometric analysis of the percentage of CD34+CD38- cells (C), performed at day 3 of culture, revealed no difference between the CTR and the 3 conditions tested. In the same way, results (D) of statistical analysis on the percentage of positive cells for lineage differentiation markers (GPA, MPO and CD14) performed by flow cytometry at day 10 of culture, showed no difference between CTR and the 3 different culture conditions.
(TIF)

Figure S2 **OBs culture conditions testing results.** Multiple mixes of media and decreasing doses of hematopoietic cytokines were tested in order to determine the mix of media capable of supporting OBs without modifying cell growth. OBs were seeded in 96-well plates (5×10^3^ cell/well) and maintained for 72 hours in 4 different culture conditions: CTR: consisting of DMEM/F12 medium supplemented with 20% FBS, COND.1: consisting of a 1∶1 mixture of IMDM and DMEM/F12 supplemented with 20% FBS, SCF (25 ng/ml), FLT3LG (25 ng/ml), TPO (10 ng/ml), IL-6 (5 ng/ml) and IL-3 (5 ng/ml), COND.2: consisting of a 1∶1 mixture of RPMI and DMEM/F12 supplemented with 20% FBS, SCF (25 ng/ml), FLT3LG (25 ng/ml), TPO (10 ng/ml), IL-6 (5 ng/ml) and IL-3 (5 ng/ml), COND.3: consisting of a 1∶1 mixture of IMDM and DMEM/F12 supplemented with 20% FBS, SCF (5 ng/ml), FLT3LG (5 ng/ml), TPO (2 ng/ml), IL-6 (1 ng/ml) and IL-3 (1 ng/ml). Cell proliferation analysis, performed by ^3^H-thymidine incorporation, demonstrated that only COND.3 does not modify cell proliferation compared to the CTR sample. Data were expressed as counts per minute± S.D.
(TIF)

Figure S3 **OBs culture conditions testing results.** Multiple mixes of media and decreasing doses of hematopoietic cytokines were tested in order to determine the mix of media capable of supporting OBs without modifying cell properties. OBs (1×10^4^/well) were seeded in 8-well chamber slides and maintained for 72 hours in 4 different culture conditions: CTR, COND.1, COND.2, COND.3. Slides were then incubated with the following anti-human monoclonal antibodies: anti-RUNX-2 (panel A), anti-osteocalcin (panel B), and anti-alkaline phosphatase (AP) (panel C). Data shown in panel B and C demonstrate that all the three different culture conditions tested do not modify osteocalcin and alkaline phosphatase expression compared to the CTR sample. On the other hand, RUNX2 expression (panel A) appears to be down-regulated in COND.1 and COND.2 compared to the CTR, whereas it appears similar in COND.3 and in the CTR sample. Our data demonstrate that COND.3 is the culture condition capable of maintaining OBs similar to CTR culture condition, therefore COND.3 has been applied in all the experiments reported in this study.
(TIF)

Figure S4 **CD34+ cell isolation from co-culture samples.** At day 3 of co-culture, CD34+ cells were separated from OBs by magnetic beads sorting, after separation cells were stained with an anti-human CD34 monoclonal antibody to assess cell purity. Data shown are representative flow cytometry of CD34+ cells before (B) and after (D) magnetic beads sorting.
(TIF)

Figure S5 **OBs isolation from co-culture samples.** At day 3 of co-culture, OBs were separated from the hematopoietic cells by magnetic beads sorting, after separation cells were stained with an anti-human CD45 monoclonal antibody to assess cell purity. Data shown are representative flow cytometry of OBs before (A, B) and after (C,D) magnetic beads sorting. Freshly isolated OBs (E) and cytospin of CD45- cells (F) were fixed for immunocytochemical analysis of Alkaline Phosphatase (AP), RUNX-2, Osteocalcin and Collagen type I (Coll.I). The percentage of positive cells was measured on 10 RGD images acquired for each marker by image analyzer (NIS-Nikon) using an objective at 10× magnification.
(TIF)

Table S1 **A**. GO categories Increased in CD34+COCULT versus CD34+Control. **B**. GO categories Decreased in CD34+COCULT versus CD34+Control.
(DOC)
