# Supplementary figures and images for: Co-Culture of Hematopoietic Stem/Progenitor Cells with Human Osteblasts Favours Mono/Macrophage Differentiation at the Expense of the Erythroid Lineage
Source: PLoS One. 2013 Jan 22;8(1):e53496. doi: 10.1371/journal.pone.0053496 (PMC3551919; doi:10.1371/journal.pone.0053496)

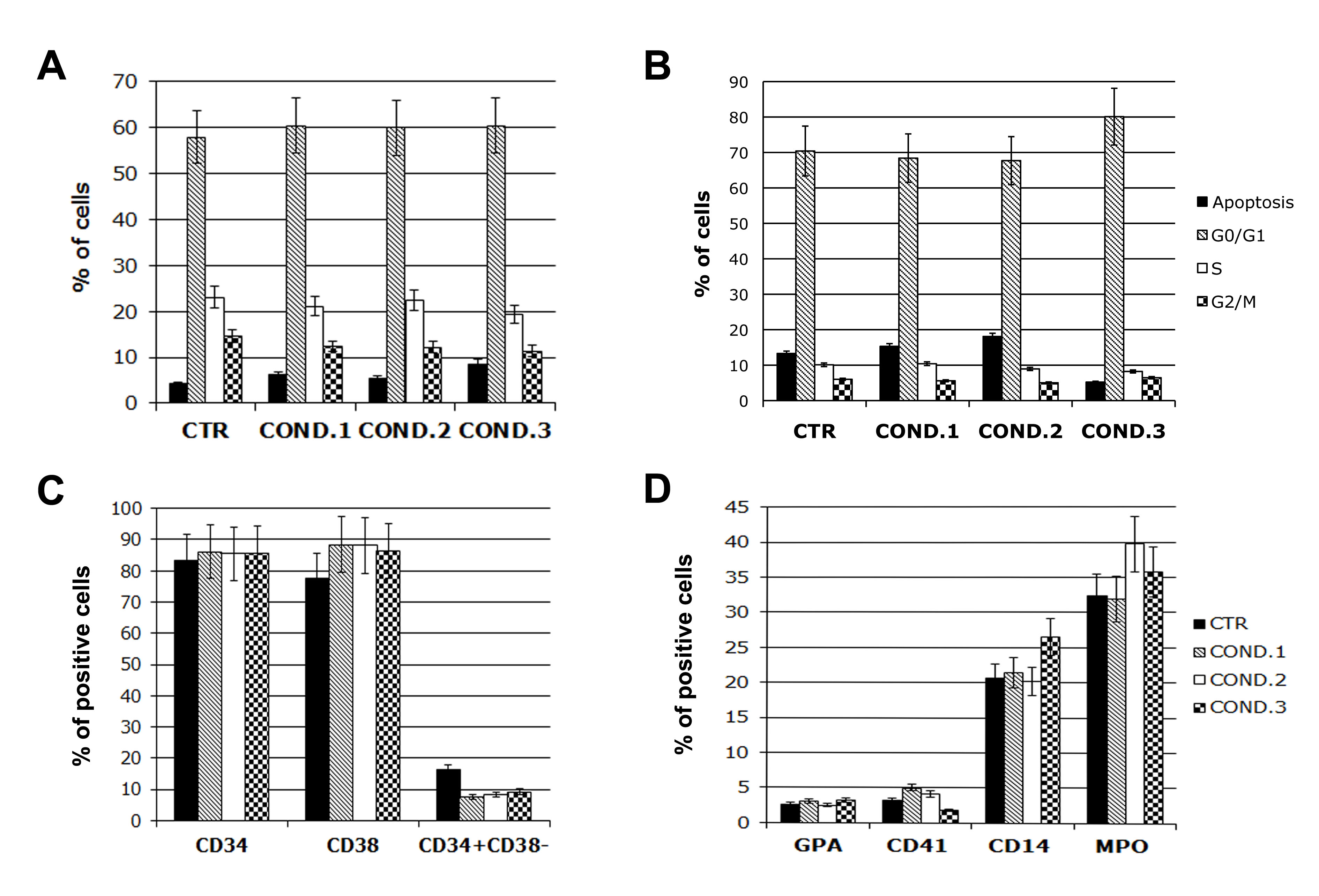

Supplement: Figure S1 — Hematopoietic cell culture conditions testing. Multiple mixes of media and decreasing doses of hematopoietic cytokines were tested in order to determine the mix of media capable of supporting CD34+ cells without modifying cell growth and differentiation properties. 24 h after isolation, CD34+ cells were seeded at a final density of 7×10^3^ cell/cm2 in 6-well plates. Cultures were maintained for 2 weeks in 4 different culture conditions: CTR: consisting of Iscove's modified Dulbecco's medium (IMDM) supplemented with 20% FBS, SCF (50 ng/ml), FLT3LG (50 ng/ml), TPO (20 ng/ml), IL-6 (10 ng/ml) and IL-3 (10 ng/ml); COND.1: consisting of a 1∶1 mixture of IMDM and DMEM/F12 supplemented with 20% FBS, SCF (25 ng/ml), FLT3LG (25 ng/ml), TPO (10 ng/ml), IL-6 (5 ng/ml) and IL-3 (5 ng/ml), COND.2: consisting of a 1∶1 mixture of RPMI and DMEM/F12 supplemented with 20% FBS, SCF (25 ng/ml), FLT3LG (25 ng/ml), TPO (10 ng/ml), IL-6 (5 ng/ml) and IL-3 (5 ng/ml), COND.3: consisting of a 1∶1 mixture of IMDM and DMEM/F12 supplemented with 20% FBS, SCF (5 ng/ml), FLT3LG (5 ng/ml), TPO (2 ng/ml), IL-6 (1 ng/ml) and IL-3 (1 ng/ml). Cell cycle analysis, performed at day 3 (A) and 7 (B) of culture by propidium iodide staining, showed no difference between the 3 culture conditions tested and the CTR sample. Flow cytometric analysis of the percentage of CD34+CD38- cells (C), performed at day 3 of culture, revealed no difference between the CTR and the 3 conditions tested. In the same way, results (D) of statistical analysis on the percentage of positive cells for lineage differentiation markers (GPA, MPO and CD14) performed by flow cytometry at day 10 of culture, showed no difference between CTR and the 3 different culture conditions. (TIF) [file pone.0053496.s001.tif]

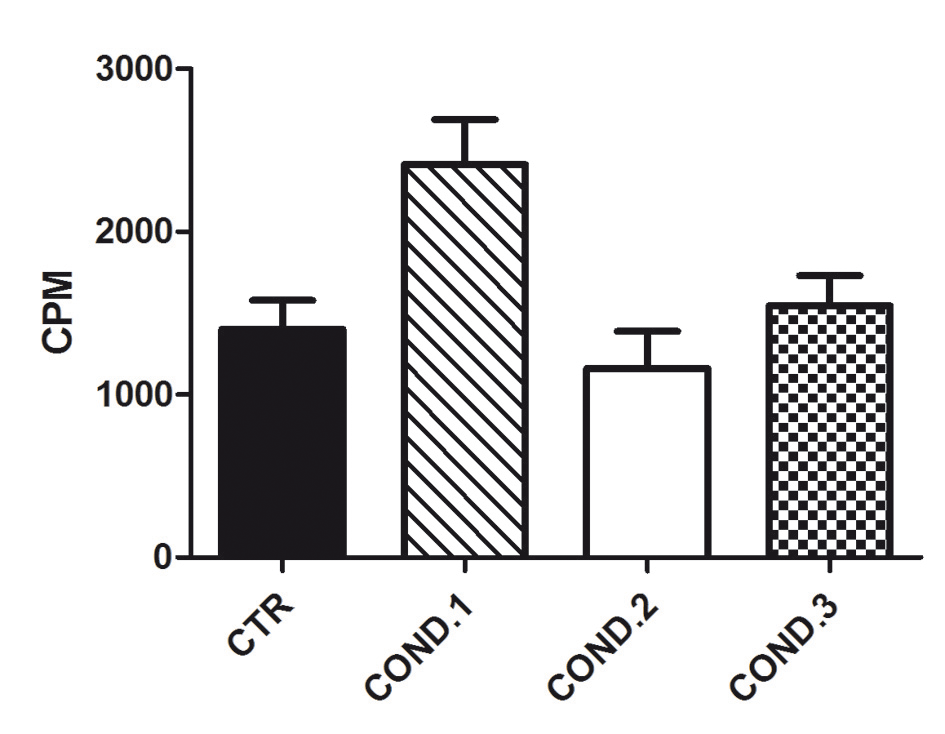

Supplement: Figure S2 — OBs culture conditions testing results. Multiple mixes of media and decreasing doses of hematopoietic cytokines were tested in order to determine the mix of media capable of supporting OBs without modifying cell growth. OBs were seeded in 96-well plates (5×10^3^ cell/well) and maintained for 72 hours in 4 different culture conditions: CTR: consisting of DMEM/F12 medium supplemented with 20% FBS, COND.1: consisting of a 1∶1 mixture of IMDM and DMEM/F12 supplemented with 20% FBS, SCF (25 ng/ml), FLT3LG (25 ng/ml), TPO (10 ng/ml), IL-6 (5 ng/ml) and IL-3 (5 ng/ml), COND.2: consisting of a 1∶1 mixture of RPMI and DMEM/F12 supplemented with 20% FBS, SCF (25 ng/ml), FLT3LG (25 ng/ml), TPO (10 ng/ml), IL-6 (5 ng/ml) and IL-3 (5 ng/ml), COND.3: consisting of a 1∶1 mixture of IMDM and DMEM/F12 supplemented with 20% FBS, SCF (5 ng/ml), FLT3LG (5 ng/ml), TPO (2 ng/ml), IL-6 (1 ng/ml) and IL-3 (1 ng/ml). Cell proliferation analysis, performed by 3H-thymidine incorporation, demonstrated that only COND.3 does not modify cell proliferation compared to the CTR sample. Data were expressed as counts per minute± S.D. (TIF) [file pone.0053496.s002.tif]

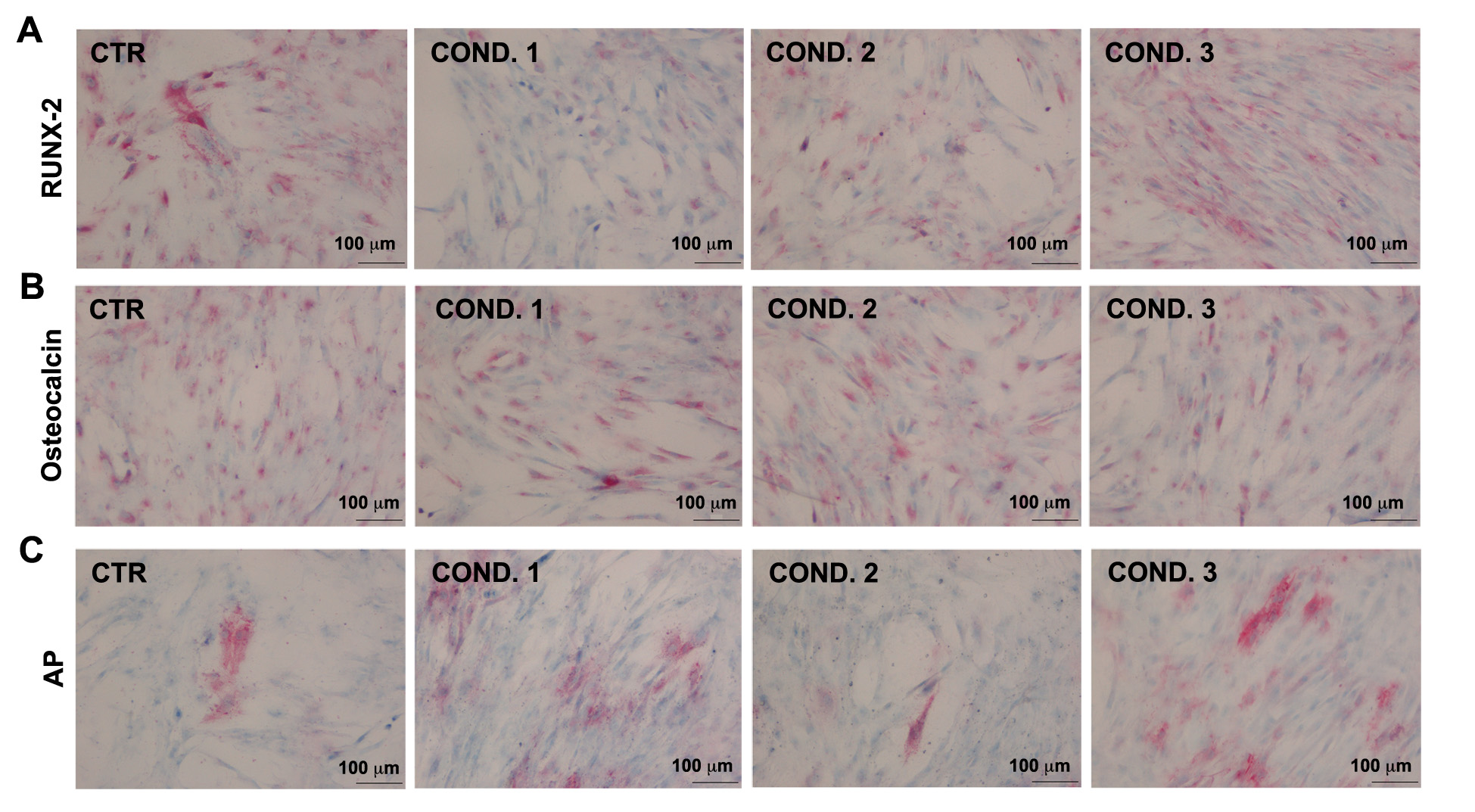

Supplement: Figure S3 — OBs culture conditions testing results. Multiple mixes of media and decreasing doses of hematopoietic cytokines were tested in order to determine the mix of media capable of supporting OBs without modifying cell properties. OBs (1×10^4^/well) were seeded in 8-well chamber slides and maintained for 72 hours in 4 different culture conditions: CTR, COND.1, COND.2, COND.3. Slides were then incubated with the following anti-human monoclonal antibodies: anti-RUNX-2 (panel A), anti-osteocalcin (panel B), and anti-alkaline phosphatase (AP) (panel C). Data shown in panel B and C demonstrate that all the three different culture conditions tested do not modify osteocalcin and alkaline phosphatase expression compared to the CTR sample. On the other hand, RUNX2 expression (panel A) appears to be down-regulated in COND.1 and COND.2 compared to the CTR, whereas it appears similar in COND.3 and in the CTR sample. Our data demonstrate that COND.3 is the culture condition capable of maintaining OBs similar to CTR culture condition, therefore COND.3 has been applied in all the experiments reported in this study. (TIF) [file pone.0053496.s003.tif]

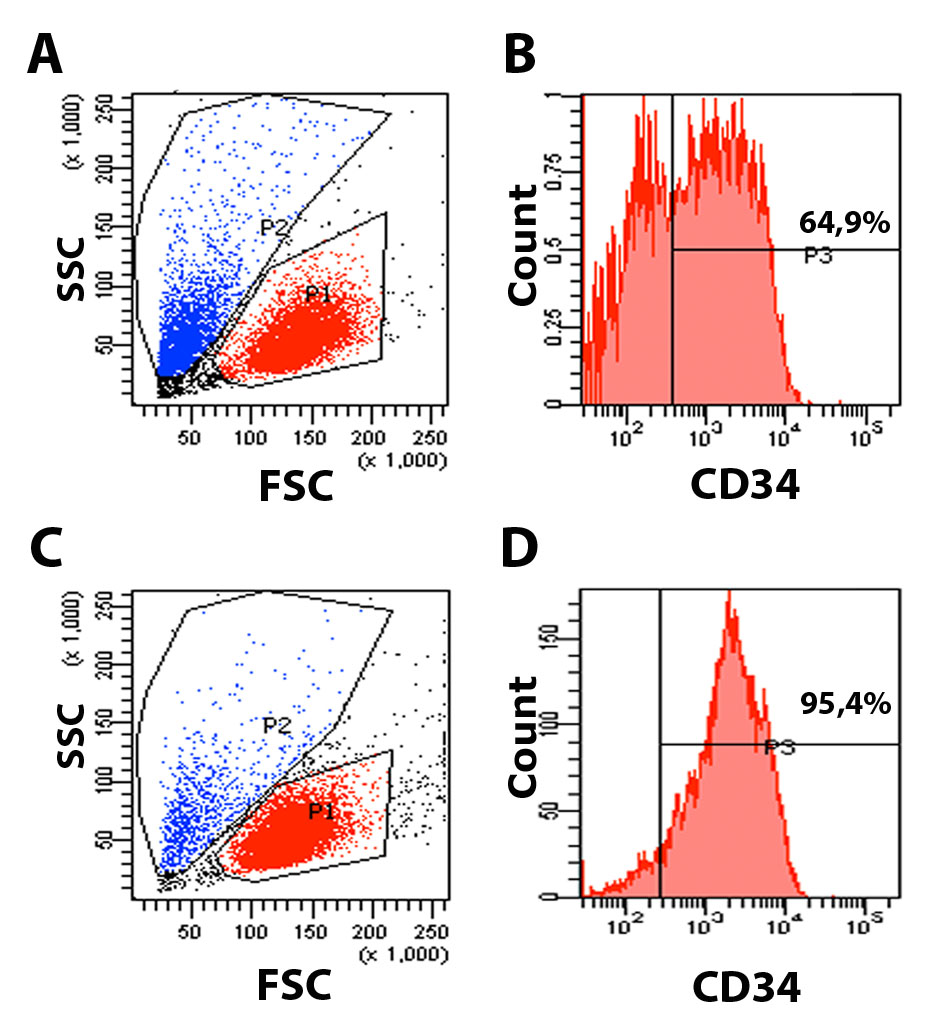

Supplement: Figure S4 — CD34+ cell isolation from co-culture samples. At day 3 of co-culture, CD34+ cells were separated from OBs by magnetic beads sorting, after separation cells were stained with an anti-human CD34 monoclonal antibody to assess cell purity. Data shown are representative flow cytometry of CD34+ cells before (B) and after (D) magnetic beads sorting. (TIF) [file pone.0053496.s004.tif]

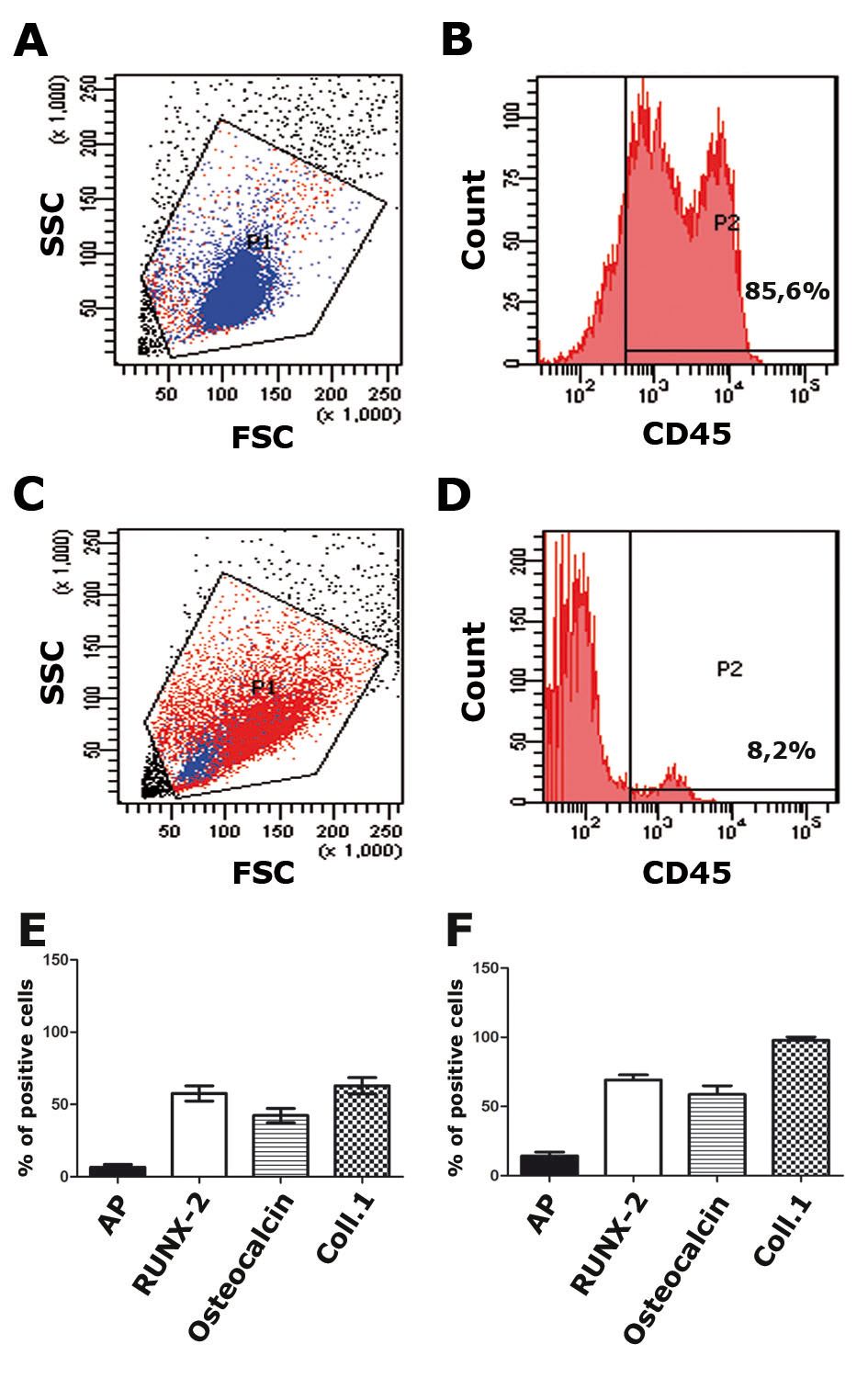

Supplement: Figure S5 — OBs isolation from co-culture samples. At day 3 of co-culture, OBs were separated from the hematopoietic cells by magnetic beads sorting, after separation cells were stained with an anti-human CD45 monoclonal antibody to assess cell purity. Data shown are representative flow cytometry of OBs before (A, B) and after (C,D) magnetic beads sorting. Freshly isolated OBs (E) and cytospin of CD45- cells (F) were fixed for immunocytochemical analysis of Alkaline Phosphatase (AP), RUNX-2, Osteocalcin and Collagen type I (Coll.I). The percentage of positive cells was measured on 10 RGD images acquired for each marker by image analyzer (NIS-Nikon) using an objective at 10× magnification. (TIF) [file pone.0053496.s005.tif]
