# Supplementary material for: Co-Culture of Hematopoietic Stem/Progenitor Cells with Human Osteblasts Favours Mono/Macrophage Differentiation at the Expense of the Erythroid Lineage
Source: PLoS One. 2013 Jan 22;8(1):e53496. doi: 10.1371/journal.pone.0053496 (PMC3551919; doi:10.1371/journal.pone.0053496)
Supplement: Table S1 — A. GO categories Increased in CD34+COCULT versus CD34+Control. B. GO categories Decreased in CD34+COCULT versus CD34+Control. (DOC) [file pone.0053496.s006.doc]

**Supplementary table 1A**. GO categories Increased in CD34+COCULT versus CD34+Control.

| **Category** | **Term** | **PValue** |
| --- | --- | --- |
| GOTERM_BP_ALL | GO:0007155~cell adhesion | 1.72E-18 |
| GOTERM_BP_ALL | GO:0022610~biological adhesion | 1.90E-18 |
| GOTERM_BP_ALL | GO:0030111 ~regulation of Wnt receptor signaling pathway | 1.47E-11 |
| GOTERM_BP_ALL | GO:0032502~developmental process | 8.69E-10 |
| GOTERM_BP_ALL | GO:0048731~system development | 1.50E-08 |
| GOTERM_BP_ALL | GO:0030198~extracellular matrix organization | 4.51E-05 |
| GOTERM_BP_ALL | GO:0043062~extracellular structure organization | 1.07E-04 |
| GOTERM_BP_ALL | GO:0030199~collagen fibril organization | 3.14E-04 |
| GOTERM_BP_ALL | GO:0009611~response to wounding | 5.27E-04 |
| GOTERM_BP_ALL | GO:0009605~response to external stimulus | 5.80E-04 |
| GOTERM_BP_ALL | GO:0031589~cell-substrate adhesion | 9.45E-04 |
| GOTERM_BP_ALL | GO:0016477~cell migration | 9.54E-04 |
| GOTERM_BP_ALL | GO:0030334~regulation of cell migration | 2.48E-03 |
| GOTERM_BP_ALL | GO:0030177~positive regulation of Wnt receptor signaling pathway | 2.44E-03 |
| GOTERM_BP_ALL | GO:0042127~regulation of cell proliferation | 3.42E-03 |
| GOTERM_BP_ALL | GO:0048870~cell motility | 4.28E-03 |
| GOTERM_BP_ALL | GO:0009888~tissue development | 4.65E-03 |
| GOTERM_BP_ALL | GO:0045655 : regulation of monocyte differentiation | 4.87E-03 |
| GOTERM_BP_ALL | GO:0007160~cell-matrix adhesion | 5.66E-03 |
| GOTERM_BP_ALL | GO:0008284~positive regulation of cell proliferation | 5.44E-03 |
| GOTERM_BP_ALL | GO:0048869~cellular developmental process | 2.20E-02 |
| GOTERM_BP_ALL | GO:0030154~cell differentiation | 2.28E-02 |
| GOTERM_BP_ALL | GO:0045785~positive regulation of cell adhesion | 3.36E-02 |
| GOTERM_BP_ALL | GO:0019827~stem cell maintenance | 6.25E-02 |
| GOTERM_BP_ALL | GO:0042221~response to chemical stimulus | 1.34E-01 |
| GOTERM_BP_ALL | GO:0001558~regulation of cell growth | 2.01E-01 |
| GOTERM_BP_ALL | GO:0051781~positive regulation of cell division | 2.32E-01 |
| GOTERM_BP_ALL | GO:0006954~inflammatory response | 3.31E-01 |
| GOTERM_BP_ALL | GO:0050793~regulation of developmental process | 4.11E-01 |

**Supplementary table 1B**. GO categories Decreased in CD34+COCULT versus CD34+Control.

| **Category** | **Term** | **PValue** |
| --- | --- | --- |
| GOTERM_BP_ALL | GO:0015669~gas transport | 4.70E-12 |
| GOTERM_BP_ALL | GO:0030218~erythrocyte differentiation | 1.55E-11 |
| GOTERM_BP_ALL | GO:0015671~oxygen transport | 5.69E-11 |
| GOTERM_BP_ALL | GO:0045648~positive regulation of erythrocyte differentiation | 0.0028207283627559236 |
| GOTERM_BP_ALL | GO:0060319~primitive erythrocyte differentiation | 0.01813426860374926 |
| GOTERM_BP_ALL | GO:0042127~regulation of cell proliferation | 0.019910432388619045 |
| GOTERM_BP_ALL | GO:0006897~endocytosis | 0.02260566844433916 |
| GOTERM_BP_ALL | GO:0010324~membrane invagination | 0.02260566844433916 |
| GOTERM_BP_ALL | GO:0070453~regulation of heme biosynthetic process | 0.02509905512542346 |
| GOTERM_BP_ALL | GO:0010724~regulation of definitive erythrocyte differentiation | 0.026534041220991404 |
| GOTERM_BP_ALL | GO:0006869~lipid transport | 0.03065248361707063 |
| GOTERM_BP_ALL | GO:0030166~proteoglycan biosynthetic process | 0.03946599462641416 |
| GOTERM_BP_ALL | GO:0010876~lipid localization | 0.03970491796886868 |
| GOTERM_BP_ALL | GO:0007165~signal transduction | 0.04674347280962729 |
| GOTERM_BP_ALL | GO:0008285~negative regulation of cell proliferation | 0.04715415418352928 |
